# The Role of Tumor-Associated Myeloid Cells in Modulating Cancer Therapy

**DOI:** 10.3389/fonc.2020.00899

**Published:** 2020-06-09

**Authors:** Christiana M. Neophytou, Chryso Pierides, Maria-Ioanna Christodoulou, Paul Costeas, Theodora-Christina Kyriakou, Panagiotis Papageorgis

**Affiliations:** ^1^European University Research Centre, Nicosia, Cyprus; ^2^Department of Life Sciences, School of Sciences, European University Cyprus, Nicosia, Cyprus; ^3^The Center for the Study of Haematological Malignancies, Nicosia, Cyprus; ^4^The Cyprus Cancer Research Institute, Nicosia, Cyprus

**Keywords:** myeloid cells, dendritic cells, macrophages, immunotherapy, Tumor-associated myeloid cells, nano-immunotherapy

## Abstract

Myeloid cells include various cellular subtypes that are distinguished into mononuclear and polymorphonuclear cells, derived from either common myeloid progenitor cells (CMPs) or myeloid stem cells. They play pivotal roles in innate immunity since, following invasion by pathogens, myeloid cells are recruited and initiate phagocytosis and secretion of inflammatory cytokines into local tissues. Moreover, mounting evidence suggests that myeloid cells may also regulate cancer development by infiltrating the tumor to directly interact with cancer cells or by affecting the tumor microenvironment. Importantly, mononuclear phagocytes, including macrophages and dendritic cells (DCs), can have either a positive or negative impact on the efficacy of chemotherapy, radiotherapy as well as targeted anti-cancer therapies. Tumor-associated macrophages (TAMs), profusely found in the tumor stroma, can promote resistance to chemotherapeutic drugs, such as Taxol and Paclitaxel, whereas the suppression of TAMs can lead to an improved radiotherapy outcome. On the contrary, the presence of TAMs may be beneficial for targeted therapies as they can facilitate the accumulation of large quantities of nanoparticles carrying therapeutic compounds. Tumor infiltrating DCs, however, are generally thought to enhance cytotoxic therapies, including those using anthracyclines. This review focuses on the role of tumor-infiltrating and stroma myeloid cells in modulating tumor responses to various treatments. We herein report the impact of myeloid cells in a number of therapeutic approaches across a wide range of malignancies, as well as the efforts toward the elimination of myeloid cells or the exploitation of their presence for the enhancement of therapeutic efficacy against cancer.

## Introduction

Immunity is the result of an intricate interaction between the innate and adaptive immune system. Innate immunity is the initial immunological response against an invading pathogen and has no immunological memory. On the other hand, adaptive immunity involves the development of immunological memory and enables the host to respond more efficiently to future exposure to the antigen. Following antigen processing, the degraded peptides associate with major histocompatibility complex (MHC) molecules within the interior of an antigen-presenting cell (APC) and are exposed on its surface. Myeloid cells, including DC and macrophages, are considered “professional” APCs. They present foreign antigens to helper T-cells on class II MHC molecules and can prime naïve T-cells. Other myeloid lineage types of cells, such as neutrophils, have no or very low expression of MHC-II and are inefficient at priming naïve T-cell responses (1). Nearly all nucleated cells can act as APCs by presenting antigens on class I MHC molecules to cytotoxic T-cells. Even though cancer cells are poor APCs, antigen presentation is involved in the body's defense against tumors.

Immune cells of various types and origins are integral components of the tumor microenvironment (TME) along with fibroblasts, endothelial cells, and extracellular components, such as collagen and hyaluronan. Cellular constituents from the lymphoid and myeloid lineage can elicit both immune suppressive and immune stimulatory functions and have an important role in regulating cancer progression and survival, as well as drug resistance (2–4). Tumors secrete factors which promote myelopoiesis and recruit circulating cells into the tumor mass, microenvironment or to secondary lymphoid organs, such as lymph nodes and spleen, and polarize their functionality to serve their survival and growth.

Hematopoietic stem cells (HSC) differentiate in the bone marrow into common myeloid progenitors (CMPs) which can give rise to DCs and to tumor-associated myeloid cells (TAMCs). TAMCs include at least four different myeloid cell populations: (a) Tumor-associated macrophages (TAMs) that exert crucial roles in regulating cancer-related inflammation, (b) monocytes that express the angiopoietin receptor Tie2 (tunica internal endothelial kinase 2) and have a pivotal function in tumor angiogenesis, (c) myeloid-derived suppressor cells (MDSCs) that can be further characterized as monocytic and granulocytic (m-MDSCs, g-MDSCs) depending on their morphology, phenotype and immune suppression functions and (d) tumor-associated neutrophils (TANs) that express pro-angiogenic factors and participate in tumor promotion (5).

**Figure 1:**
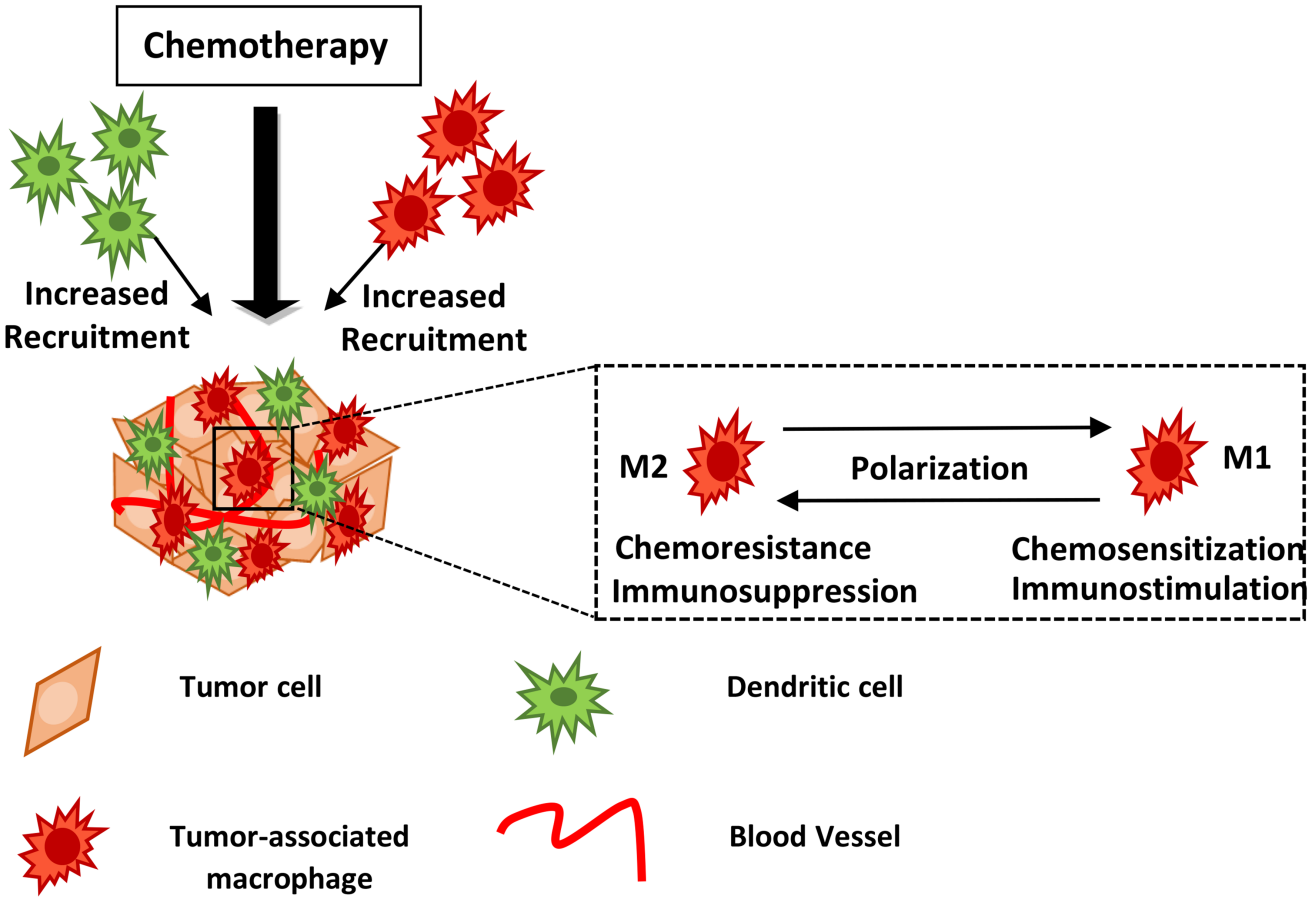
Myeloid cells may block or facilitate chemotherapy. Chemotherapy induces recruitment of innate immune cells including macrophages and dendritic cells into the treated tumor tissue. Drug treatment may lead to TAM polarization from the M2- to an M1-like phenotype, hindering tumor growth and metastasis. The mechanisms of action utilized by myeloid cells in supporting or blocking chemotherapy are described in the text and summarized in Table 1. DCs, Dendritic cells; TAMs, Tumor-associated macrophages.

Ideally, a competent immune system recognizes tumor-specific and embryonic antigens; however, cancer cells manage to escape immune surveillance by secreting immune escape variants, recruiting myeloid-derived cells and either maintaining them in an immunosuppressive phenotype or polarizing them into tumor-promoting cell types (6, 7). The mechanisms of recruitment of myeloid cells to the TME are often the targets of anti-cancer therapy. Emerging evidence indicates that TAMCs interfere with or facilitate most therapeutic approaches, including conventional chemotherapy, targeted approaches, radiotherapy and immunotherapy. TAMCs are found abundantly in the tumor stroma; high density of TAMCs has been significantly associated with poor prognosis in several cancer types including head and neck, breast, thyroid, liver, kidney, pancreatic, bladder, endometrial, ovarian, oral cancer, as well as Hodgkin lymphoma (8–10). Many studies have shown that TAMCs can induce chemoresistance against first-line chemotherapeutic drugs (Figure 1). In this review, we discuss the interplay between myeloid cells, mainly focusing on TAMs, MDSCs and DCs, and cancer therapy, the mechanisms of action by which they exert either positive or negative effects as well as provide insights related to current controversies in the field.

## Mechanisms of Recruitment and Functions of Myeloid Cell Populations in the Tumor Microenvironment

Cell populations of myeloid origin are critically important components of the TME as they play a central role in the regulation of anti-tumor immune responses. At the same time, inflammatory immune cells such as tumor-infiltrating lymphocytes (TILs), natural killer (NK) cells, NK T-cells, and B cells are also engaged within the TME, which further interact to affect the growth and function of cancer cells. While myeloid cells are required for enabling anti-tumor immunity, they can also have an immunosuppressive role in established tumors by promoting immune evasion, and facilitating primary tumor growth, progression and metastasis (11).

Colony-stimulating factor-1 (CSF-1) and colony-stimulating factor-2 (CSF-2), also known as macrophage colony-stimulating factor (M-CSF) and granulocyte macrophage colony-stimulating factor (GM-CSF), respectively, are secreted cytokines that regulate mature myeloid cell populations by affecting their activation, survival, mobilization and differentiation. They have also been implicated in the development of many diseases, including in tumor progression and metastasis (12). Cancer cells expressing high levels of M-CSF, recruit TAMs to the tumor site, *via* their receptor CSF-1R (13). The elevated expression of M-CSF in tumors, and consequently the presence of CSF-1R-positive macrophages, has been correlated with poor prognosis in patients with breast, bladder and ovarian cancer (9). M-CSF induces high expression of C-C motif chemokine ligand 2 (CCL2) by macrophages, a chemokine that acts as a chemoattractant driving them to the tumor but may also affect their polarization and survival (14, 15). Since M-CSF also mediates the polarization of macrophages to the tumor-promoting type (16), the targeting of the M-CSF/CSF-1R axis, represents an attractive therapeutic approach and has shown efficacy in cancer metastasis models and in several murine models of cancer (17–20).

A combination of cytokines, particularly granulocyte colony-stimulating factor (G-CSF) or GM-CSF, interleukin (IL)-6, and the transcriptional regulator CCAAT/enhancer-binding protein (C/EBP) are required for the differentiation of bone marrow progenitors into MDSCs (21, 22). Whilst solid indications demonstrate that MDSCs directly suppress cytotoxic leukocytes, conventional and plasmacytoid dendritic cells (pDC) can also have immunoregulatory effects in tumors (23). Consequently, a more comprehensive characterization of these subsets and a better understanding of their recruitment and expansion mechanisms are of paramount importance for the development of novel cancer therapeutic strategies as well as for the potential improvement of existing ones.

DCs are essential for the cross-priming of cytotoxic T lymphocytes against tumor-specific antigens; however tumor-residing DCs can cause cell anergy and tolerance by expressing low levels of costimulatory molecules and pro-inflammatory cytokines (24). TAMs that have a classic (M1) activation state are characterized by anti-tumor immunity, proinflammatory activity and the induction of T-cell responses (25, 26). The presence of M1-type macrophages in high numbers within the TME, has been associated with good prognosis in patients with non-small cell lung cancer (NSCLC), colorectal, hepatocellular, ovarian and gastric cancer (27). In malignant tumors, TAMs resemble M2-type macrophages, which undergo alternative (M2) activation. These cells have the ability to support tumor growth, inhibit immunity against the tumor, and promote tissue repair (28). These have been generally considered as a promising target for tumor therapy, with studies concentrating on the inhibition of macrophage recruitment, survival, and tumor-promoting activity in tumors, as well as, predominantly, on the shift of tumor-promoting M2 TAMs toward tumor-suppressive M1-type macrophages (29).

The importance of myeloid cells in facilitating the killing of tumor cells has been highlighted by many studies (30, 31). Myeloid cells can exert significant anti-tumor functions by activating NK and CD8^+^ T-cells. Cancer cells can be detected by NK cells through the expression of ligands for the receptor NKG2D (32). The binding of these ligands serves as a major signal of activation NK cells to stop aberrant cell proliferation and can be further enhanced through the function of myeloid cells. In fact, macrophages and DCs express Dectin-1, a receptor that recognizes N-glycan structures found on the surface of certain types of tumor cell. Activation of Dectin-1 induced a signaling pathway that directs the activity of NK cells against the tumor in a lung metastasis model of B16F1 melanoma cells (33). In addition, the expression of calreticulin on the surface of cancer cells can be recognized and processed by macrophages which then activate CD4^+^ and CD8^+^ T-cells. T-cells can then produce interferon gamma (IFN-γ) to induce cytolysis in cancer cells (34).

At the same time, tumor cells take advantage of the ability of myeloid cells to inhibit tumor-targeting immune responses and to mediate immunosuppressive effects. Tumor growth and progression is restrained to genetic or epigenetic alterations which, in turn, affect tumor development and invasion into the surrounding tissues. During this process, cancer cells reprogram infiltrating stromal cells to support an abnormally regulated inflammation that is hyporesponsive to the tumor (35). Cancer cells achieve this by producing immune effector molecules, such as tumor necrosis factor-α (TNFα) and interleukin-6 (IL-6), growth factors that regulate tumor proliferation and angiogenesis, such as transforming growth factor-β (TGF-β) and vascular endothelial growth factor (VEGF), and matrix metalloproteinases (MMPs) that degrade extracellular matrix proteins (31, 36). Abundance of tumor-infiltrating and circulating monocytes, MDSCs and neutrophils, is associated with advanced cancer progression, decreased disease-free and overall survival (37).

MDSCs can be subdivided into two major groups: monocytic MDSCs (m-MDSCs) and granulocytic MDSCs (g-MDSCs) that are morphologically similar to monocytes and granulocytes, respectively. In humans, m-MDSC have the same density fraction as monocytes. However, monocytes express the MHC class II cell surface receptor HLA-DR in high levels while m-MDSCs are characterized by low or no HLA-DR expression. Furthermore, m-MDSCs have a CD11b^+^HLA-DR^−^CD14^+^CD15^−^ phenotype (38). The expansion of m-MDSCs is induced by a combination of soluble factors produced by tumor and/or surrounding cells such as stromal cells, T-cells or macrophages including VEGF, GM-CSF, M-CSF, IL-4, IL-6, IL-10, prostaglandin E2 (PGE2), MMP9, C-X-C motif chemokine ligand 5 (CXCL5), and CXCL12/ stromal-derived factor 1 alpha (SDF1-alpha) (39). Human g-MDSCs are phenotypically characterized as CD11b^+^HLA-DR^−^CD14^−^CD15^+^ (38).

In addition to their morphological and phenotypic differences, m-MDSCs and g-MDSCs also have different mechanisms by which they suppress immune function. TAMs and m-MDSCs have a shared mechanism for the expression of inducible nitric oxide synthase (iNOS) and arginase. Indoleamine 2,3-dioxygenase (IDO), inducible nitric oxide synthase (iNOS), arginase I and effector cytokine production have been proposed to be involved in suppression of T-cell proliferation and cytotoxicity (40). iNOS generates nitric oxide (NO) causing the inhibition of IL-2 receptor signaling, blocking T-cell activation and proliferation, thus leading to an immunosuppressive effect (41). TAMs that have a classic (M1) activation state (25), paradoxically express iNOS, whose immunosuppressive effect is, however, overawed by other proinflammatory and anti-tumor mediators. TAMs and MDSCs commonly employ another immunosuppressive mechanism involving the generation of reactive oxygen species (ROS) and reactive nitrogen species, such as peroxynitrite mediated by iNOS and arginase (42). Following T-cell receptor (TCR) modification of anti-tumor T-cells due to peroxynitrite, they become unable to bind to their equivalent MHC-peptide antigens presented by APCs in the tumors (43). Furthermore, m-MDSCs can indirectly suppress anti-tumor immunity, through the production of TGF-β and IL-10 cytokines, which inhibit anti-tumor TILs, generate regulatory T-cells (Tregs) in the tumor and induce DCs into a regulatory phenotype (44). Alternatively, immunosuppressive g-MDSCs share many of the immunosuppressive mechanisms of m-MDSCs, but they also produce ROS, which are able to alter the TCR of TILs through direct cell-to-cell contact (45, 46).

Understanding the interactions between the tumor and infiltrating cells will allow the prediction of tumor progression as well as the design of novel anticancer therapies which will target the tumor microenvironment. Working on the basis that cancer and myeloid cells use common pathways for immune system regulation, along with the fact that myeloid cells have the ability to network with different immune cell populations toward inducing an anti-tumor immune response, myeloid-based therapies have increasingly gained attention as possible adjuncts to improve efficacy of current therapies, including immune checkpoint inhibitors (ICIs), oncolytic viruses, dendritic cell vaccines, and traditional chemoradiation. Characterizing the myeloid compartment also allows for patient stratification on prognosis and response to immunotherapy based on the presence of myeloid-specific biomarkers in combination with tumor mutational burden, checkpoint expression, and T-cell receptor diversity (47).

## TAMCs In Conventional Chemotherapy and Targeted Therapy

### Negative Impact of TAMCs in Chemotherapy

The presence of TAMs in the TME provides cancer cells with cytokines, growth factors and proteases that mediate survival, chemoresistance and promote invasion. One example is cysteine cathepsin proteases that are produced by macrophages and have been shown to enhance pancreatic tumor growth and invasion (48). Paclitaxel, an anti-microtubule agent belonging to the Taxane family, is used for the treatment of ovarian, breast and non-small cell lung cancer. Following Paclitaxel treatment, the infiltration of macrophages in mammary tumors as well as cathepsin levels were increased. In co-culture experiments, macrophages protected cancer cells from Paclitaxel treatment by producing cathepsins B and S; this was reversed by cathepsin inhibition, suggesting that concurrent inhibition of TAMs along with chemotherapy may limit the development of resistance (49). The chemoresistance facilitated by macrophages was also observed against Etoposide and Doxorubicin (49).

As previously mentioned, CSF-1 and its receptor (CSF-1R) are also involved in the tumor-promoting functions of TAMs. The blockade of CSF-1 decreased macrophage infiltration and improved response of mammary and pancreatic carcinomas to chemotherapy (50). B-Raf is a serine/threonine protein kinase, that acts downstream of RAS and has an important role in the mitogen-activated protein kinase (MAPK)/extracellular signal-regulated kinase (ERK) signaling pathway. The *BRAF* gene is commonly mutated in melanoma resulting in a constitutive function of the B-Raf protein (51). In a mouse model of melanoma, the concurrent treatment with CSF-1R inhibitor, PLX3397, and BRAF inhibitor, Vemurafenib, resulted in enhanced anti-tumor responses attributed to a significant reduction of tumor-infiltrating myeloid cells and an increase of tumor-infiltrating lymphocytes (52). Another specific CSF-1R inhibitor, GW2580, in combination with an anti-VEGFR-2 antibody, synergistically inhibited tumor angiogenesis in lung cancer and melanoma *in vivo* models; blocking CSF-R1 led to reduced tumor recruitment of TAMs and reverted a TAM-mediated compensatory antiangiogenic mechanism involving MMP-9 (53). The chemoresistance induced by tumor-infiltrating macrophages could also be mediated by the expression of IL-10; therapeutic blockade of IL-10 receptor (IL-10R) had similar effects to CSF-1 neutralization and enhanced tumor response to Paclitaxel and Carboplatin in the MMTV-PyMT transgenic model of luminal B-type mammary carcinoma (54).

In two human hepatocellular carcinoma xenograft mouse models (HCCLM3-R and SMMC7721) tumor growth, lung metastasis, and tumor angiogenesis were observed following treatment with Sorafenib, a multi-kinases inhibitor. Sorafenib caused a significant increase in macrophage peripheral recruitment and intratumoral infiltration accompanied with elevation of CSF-1R, CXCL12/SDF-1 alpha, and VEGF. SDF-1/CXCL12 has been correlated with cancer cell invasion by recruiting macrophages to the area surrounding the tumor (55). Targeting of macrophages using two specific drugs, Zoledronic acid (ZA) and Clodrolip, in combination with Sorafenib significantly hindered tumor progression, angiogenesis, and metastasis to the lungs compared with animals treated with Sorafenib alone (56). Serial low doses of Sorafenib augmented tumor inhibition and function of CD8^+^ T-cells by decreasing MDSCs and reversing the immunosuppressive microenvironment in an E.G7/OT-1 murine model (57).

The importance of TAM polarization is evident in patients treated with platinum-containing chemotherapy. Chemoresistance is associated with elevated levels of PGE2 and IL-6, two inflammatory mediators that are regulated by cyclooxygenase (COX), drive differentiation of monocytes to the M2 tumor-promoting phenotype. Treatment with Cisplatin or Carboplatin increased the potency of cervical and ovarian cell lines to induce M2 macrophages that produce IL-10. Tumor-produced IL-6 and PGE2 led to increased levels of activated Signal Transducer and Activator of Transcription 3 (STAT3) and decreased levels of activated STAT1 and STAT6, respectively. Blockade of canonical Nuclear Factor Kappa-light-chain-enhancer of activated B cells (NF-κB) reduced the production of PGE2 and/or IL-6 by the tumor cells and abrogated the effect of the chemotherapy. Blocking COX using the specific inhibitor indomethacin as well as inhibition of interleukin-6 receptor (IL-6R) with the clinical monoclonal antibody Tocilizumab, prevented M2 differentiation. These results propose that chemoresistance may be caused *via* an increase in the number of M2 macrophages and that concurrent therapy with COX inhibitors and/or anti-IL-6R antibodies might facilitate platinum-based chemotherapy in resistant tumors (58).

Studies have shown that B and T lymphocytes may exert pro-tumor effect by regulating the activity of myeloid cells, resulting in resistance to therapy and promoting metastasis in different malignancies, including epithelial hyperplasia, squamous carcinomas and prostate cancer (59–61). In the absence of a robust CD8^+^ CTL response, CD4^+^ T-effector lymphocytes enhance breast cancer metastasis to the lung by enhancing the activity of TAMCs (62). In a study using an aggressive transgenic mouse model of mammary adenocarcinoma development [MMTV–polyoma middle T (PyMT) mice (63)], combination of CSF1R-signaling antagonists that block infiltration of mammary tumors by CD68^+^ macrophages, in combination with Paclitaxel, improved survival, delayed primary tumor growth and reduced pulmonary metastasis. This study also showed that the presence of an enriched CD68^high^ /CD4^high^ /CD8^low^ cell population, significantly correlates with reduced overall survival (OS) for patients with breast cancer (64).

Depletion of myeloid-lineage cells enhanced anti-cancer immunity associated with gemcitabine (GEM) treatment in mice with pancreatic ductal adenocarcinoma (PDAC) tumors (65). GEM is a nucleoside analog used as first-line treatment. Myeloid cells expressing the granulocytic marker (GR-1) were found in abundance in PDAC tumor tissues while CD4^+^ and CD8^+^ cells were present in small numbers. Following GEM treatment, myeloid cells in tumor tissues and in peripheral blood decreased while numbers of CD4^+^ or CD8^+^ cells increased suggesting that anti-cancer immunity was enhanced. In addition, concurrent treatment of mice with GEM and further depletion of myeloid cells using an anti-GR-1 antibody significantly prolonged survival (65). Inflammatory breast cancer (IBC) is often characterized by overexpression of epidermal growth factor receptors 1 and/or 2 (ErbB1, ErbB2) that activate downstream survival pathways phosphatidylinositol-3-kinase (PI3K)/protein kinase B (AKT) and MAPK (66). Lapatinib (a dual ErbB1/2 tyrosine kinase inhibitor) is for IBC patient treatment and functions by blocking ErbB1 and ErbB2 receptor phosphorylation and activation (67). Following combination treatment with Lapatinib and the anthracycline Doxorubicin in an MMTV-neu mice HER2-positive breast cancer model, CD8^+^ T-cells secreting IFN-γ contributed to the anti-tumor effects of these drugs. Increased effectiveness correlated with decreased content of immunosuppressive TAMs in the tumor bed induced by Doxorubicin (68).

In PDAC patients, TAMs may contribute to resistance to GEM by reducing GEM-induced apoptosis. *In vitro* co-culture of macrophages with cancer cells significantly reduced of Caspase-3 activation and apoptosis during GEM treatment. In *in vivo* PDAC models of mice, macrophages recruitment to the tumor using CSF1R-antagonist GW2580, enhanced the effect of GEM; the presence of TAMs in the tumor seems to convey resistance to GEM by inducing upregulation of the enzyme cytidine deaminase (CDA). CDA metabolizes GEM following its transfer into the cell. In PDAC cells, decreasing the expression of CDA inhibited the protective effect of TAMs against GEM (69).

TAMs have also been found to confer resistance to MAPK pathway inhibitors against melanoma. The mechanism of action involved expression of TNFα by TAMs and acted through the lineage transcription factor microphthalmia transcription factor (MITF). MITF plays a key role in melanocyte differentiation by transcriptional control of genes expressing enzymes involved in melanin synthesis; in addition, MITF has protumoral targets including B-cell lymphoma 2 (Bcl-2) and Hypoxia-Inducible Factor 1 (HIF-1) that convey survival signals (70). TNF binding to TNFR activates multiple signaling pathways, including MAPK and NF-κB and induces apoptosis and necroptosis pathways (71). Inhibition of TNFα signaling with IκB kinase inhibitors significantly improved the effectiveness of MAPK pathway inhibitors by targeting not only the melanoma cells but also the tumor microenvironment (72). Also, in melanoma cells macrophages conferred resistance to BRAF inhibitors in mouse and human tumor models, which was overcome by blocking the MAPK pathway or VEGF signaling. The presence of macrophages predicts early relapse following therapy in melanoma (73). Administration of the BRAF small molecule inhibitor PLX4720 had similar effects in a murine model of melanoma; PLX4720 reduced tumor growth by promoting the formation of a more immune stimulatory microenvironment correlated with a reduced accumulation of CD11b^+^/GR-1^+^ myeloid cells (74).

The polarization of macrophages into M1 or M2, has important implications for therapeutic strategies in human cancers (Figure 1). The M2 subtype is thought to support tumor growth. In a spontaneous mouse model of gastrointestinal stromal tumor (GIST) as well as upon analysis of freshly procured human GISTs, TAMs displayed an M1-like phenotype and function at baseline; however, treatment with Imatinib, that acts as a KIT oncoprotein inhibitor, induced TAMs to become M2-like, in both mice and humans. This process involved the interaction of TAMs with apoptotic tumor cells leading to the induction of C/EBP transcription factors and development of resistance to Imatinib (75). Re-programming of macrophages from an immune-inhibitory M2-like subtype toward an immune-stimulatory M1-like subtype, by targeting the VEGF receptor 2, enhanced anticancer efficacy in a CD8^+^ T-cell–dependent manner in murine breast cancer models, suggesting that combination of anti-angiogenic therapy with other types of drugs may facilitate anti-tumor effects by altering the phenotype of TAMs (76).

### TAMCs Facilitate Chemotherapy

#### Tumor Associated Macrophages

Common chemotherapeutic drugs may act as alkylating agents. They function by adding an alkyl group to the guanine base of the DNA molecule, making the strands unable to uncoil and separate and causing breakage of the DNA strands and apoptosis. In response to alkylating agents, an immune response involving the activation of macrophages is initiated and this involves the high mobility group box 1 (HMGB1) protein. HMGB1 is an important chromatin protein that bends DNA, facilitates protein binding and helps regulate transcription (77). Activated macrophages and monocytes secrete HMGB1 which acts as a mediator of inflammation (78). In an athymic mouse tumor xenograft model where tumors were formed using immortalized murine embryonic fibroblasts (MEFs) overexpressing Bcl-xl, DNA alkylating therapy led to inhibition of protumor cytokines such as IL-4, IL-10, and IL-13, recruitment of innate immune cells including neutrophils, NK cells and macrophages into the treated tumor tissue and to complete tumor regression; loss of HMGB1 resulted in increased levels of protumor cytokines upon treatment and failure to activate innate immunity (79).

Contradictory to the chemoresistance developed by macrophages following exposure to Paclitaxel as described in the previous chapter, other studies suggest that the agent can promote anti-tumor immunity by polarizing M2 macrophages to the M1-like phenotype (80). As previously mentioned, macrophages that polarize as M1-type are considered pro-inflammatory and potentially mediate anti-tumor activities, whereas those that polarize as M2-type decrease inflammation and may promote cancer cell growth *via* angiogenesis and immunosuppression (81–83). Similarly, administration of Doxil nanomedicine combined with the TGFβ inhibitor Tranilast, increases immunostimulatory M1-type macrophage content over the M2-type in mouse models of triple-negative breast cancer (84). A possible explanation, which may also be applied to other observations, could be the improved blood vessel perfusion, oxygenation, and normalization of the TME. Moreover, macrophages polarize to the M1-type following exposure to IFN-γ and Lipopolysaccharide (LPS) (85). Paclitaxel induced TAMs toward an M1-like profile in mouse models of melanoma and breast tumors which was depended on the presence of Toll-Like receptor 4 (TLR4) on myeloid cells. Absence of TLR4 weakened the antitumor effect of Paclitaxel. This was confirmed using gene expression analysis of tumor samples from ovarian cancer patients that showed enrichment of genes correlated to the M1 macrophage activation profile following Paclitaxel treatment (80). A similar effect was observed using nanoparticles loaded with albumin-bound paclitaxel (nAb-PTX) (86). Using 3D-spheroid models of co-cultured breast cancer cells with macrophages as well as *in vivo* models, researchers showed increased drug accumulation within the macrophages and the tumor spheroids; this shifted the TME toward a pro-inflammatory, anti-tumorigenic state. In addition, the loaded nanoparticles (NPs) increased macrophage motility and delivery of the NPs toward the cancer cells promoting apoptosis and inhibiting proliferation. Importantly, the NPs loaded with PTX induced macrophage differentiation toward the anti-cancer M1 phenotype (86).

Following surgery, the OS of gastric carcinoma patients treated with 5-fluorouracil-based chemotherapy was positively correlated with increased numbers of CD68^+^ macrophages (87). In a cohort study involving 110 patients with PDAC, post-surgical adjuvant chemotherapy was shown to “re-educate” TAMs and help them elicit an anti-tumoral response. Cyclophosphamide (CTX) is a common chemotherapeutic agent, which acts as an alkylating agent, that is used for the treatment of several human malignancies (88). Enrichment of TAMs at the tumor–stroma interface positively correlated with responsiveness to CTX therapy in patients with PDAC, independently of the density of T-cells. A similar effect was observed *in vitro*, where in the presence of GEM, macrophages activated a cytotoxic gene expression program and switched to an anti-tumor phenotype (89). In patients with invasive ductal breast cancer receiving adjuvant multimodal chemotherapy, tumor infiltration by macrophages correlated with improved time-to-relapse (TTR) and OS (90). Similar observations were made in patients with colorectal carcinoma (CRC). In a study of 1,400 CRCs patients treated with adjuvant multimodal chemotherapy, the level of CD16^+^ macrophage infiltration correlated with that of CD3^+^ and CD8^+^ lymphocytes and with improved survival compared with patients with low infiltration (91). These results contradict with previous reports indicating that a low number of CD68^+^ macrophages infiltrates associates with improved patient survival (64); the role of TAMs in predicting patient response to treatment is therefore complex and remains to be further elucidated.

TAMs may also act as a slow-release reservoir for therapeutic nanoparticles (TNPs). TNPs are generally applied as a vehicle to deliver drugs specifically to the tumor site and increase their accumulation. TNPs comprised by a fluorescent platinum (IV) pro-drug and a polymer platform (PLGA-b-PEG) were shown to accumulate in TAMs. TAMs acted as a local drug depot and allowed for the slow release of the DNA-damaging drug to neighboring tumor cells. The depletion of TAMs led to a decrease of intratumoral TNP accumulation and treatment efficacy in a lung cancer animal model. The presence of TAMs can therefore affect the design and use of TNPs for tumor targeting (92).

Histidine-rich glycoprotein (HRG), is a host-produced immunomodulatory and antiangiogenic factor that regulates tumor vessel formation and inflammation. HRG is produced in the tumor stroma from plasma or platelets and has been reported to inhibit tumor growth and metastasis and to enhance chemotherapy in brain tumor models (93, 94). This effect is mediated through downregulation of placental growth factor (PlGF) followed by polarizing TAMs from the M2- to a tumor-inhibiting M1-like phenotype. It is likely that HRG/PIGF/M1-type TAMs enhance the antitumor immune response and facilitate vessel normalization, effects known to hinder tumor growth and metastasis and to facilitate chemotherapy (29).

#### Dendritic Cells

The therapeutic efficacy of anthracyclines, a group of conventional chemotherapeutic drugs that act by inhibiting topoisomerase II causing DNA damage, may depend on the presence of intratumoral dendritic cells. Specifically, mice bearing fibrosarcomas were treated with the anthracycline mitoxantrone (MTX). This caused cancer cell death which led to the release of Adenosine triphosphate (ATP), recruitment of myeloid cells and their differentiation locally into inflammatory DC-like cells. The presence of the DC-subset was responsible for the immune system-depended anti-tumor effect of anthracycline, by engulfing tumor antigens and presenting them to T-lymphocytes. Importantly, preventing tumor infiltration by myeloid cells, abolished the anti-tumor immune response following chemotherapy (95). The activation of autophagy in cancer cells is essential for increasing the recruitment of DC and improve the efficacy of chemotherapy. In mouse models of colorectal cancer and sarcomas, response to chemotherapy led to the release of ATP by autophagy-competent cancer cells, attracted dendritic cells and T lymphocytes into the tumor bed and restored chemotherapeutic responses (96). Exposure to chemotherapeutic agent Paclitaxel, does not significantly affect the viability of DCs in concentrations up to 100 μM (97). Furthermore, exposure of DCs to clinically relevant concentrations of Paclitaxel led to increased HLA class II expression which was similar to the expression observed when DCs are exposed to lipopolysaccharide (LPS). Paclitaxel also increased proliferation of allogeneic T-cells. This study suggests that Paclitaxel may induce immunostimulatory effects in certain concentrations and may find clinical applications in patients receiving DC vaccines (97).

DC-like cells are important in the anti-tumor immune response since they have enhanced abilities to activate CD8^+^ T-cells compared to TAMs. The effect of Oxaliplatin combined with Cyclophosphamide (Oxa-Cyt) treatment on tumors relied on TLR4 signaling; Oxa-Cyt treatment led to an increase of TLR4 selectively in DC cells within the tumor stroma and ultimately led to CD8^+^ T-cell anti-tumor immunity in lung adenocarcinoma mouse models (98). CTX has been shown to induce anti-cancer effects by stimulating immunomodulatory factors; in patients with hematologic malignancies, a single high-dose treatment with CTX induced an increase in the number of DCs (99). DC turnover in the spleen, liver, and tumor site as well as their expansion in the circulation, enhanced the beneficial anti-tumor effects of CTX in mice models. The expansion of DC (CD11c^+^CD11b^+^) induced by CTX was associated with proliferation of DCs in the bone marrow (BM) prior to their increase in the circulation in a melanoma mouse model (100). These newly recruited DCs secreted more IL-12 and less IL-10 compared with those from untreated animals and were able to induce anti-tumor T-cell responses in a colon cancer model (101).

In addition to facilitating chemotherapy, DCs may also contribute to specific cancer targeting induced by small molecule inhibitors. Overactivation of the Jak2/STAT3 signaling pathway induced by tumor-derived factors may be responsible for irregular DC differentiation and function in colon cancer (102). The use of a selective inhibitor of Jak2/STAT3, JSI-124, led to activation of the transcription factor NF-κB, promoted the differentiation of mature DCs and led to T-cell activation (103). JSI-124 has been previously shown to inhibit the growth of tumors with constitutively active STAT3 (104).

Finally, recent evidence suggest that dosing and scheduling of chemotherapy administration may also regulate anti-tumor immunity. In contrast to traditional chemotherapy protocols in which the anti-cancer agents are cyclically administered near the maximum tolerated dose (MTD) alternated with longer drug-free periods, metronomic chemotherapy protocols suggest a more frequent administration of doses as low as 1/10th of the MTD (105). Besides significantly reducing drug-mediated adverse effects, metronomic chemotherapy may enhance anti-tumor immune responses. More specifically, metronomic administration of CTX was found to increase infiltration of DCs, macrophages and NK cells in mouse models as well as end-stage patients of various cancer types (106, 107). In addition, metronomic chemotherapy regimens may also promote vascular normalization to enhance delivery of co-administered drugs, thus further improving the efficacy of anti-cancer treatments (108, 109). The mechanisms of action utilized by myeloid cells in supporting or hindering chemotherapy are summarized in Table 1.

**Table 1:** Proteins implicated in TAM negative or positive contribution to chemotherapy.

**Drug**	**TAMs—Mechanism of action**	**Negative contribution**
Sorafenib	CSF-1R↑, CXCL12/SDF-1α↑, VEGF↑	Cancer cell invasion Chemoresistance
Carboplatin OR Cisplatin	STAT3↑, IL-6↑ and PGE2↑, STAT1↓, and STAT6↓	Chemoresistance via M2↑ Macrophages
Paclitaxel Etoposide Doxorubicin	Cathepsin B and S↑	Apoptosis inhibition Cancer cell protection Chemoresistance
Gemcitabine (GEM)	Caspase-3↓ activation, CDA enzyme↑	GEM-induced apoptosis inhibition Chemoresistance
	**TAMs and DC—Mechanism of action**	**Positive contribution**
Alkylating agents	HMGB1↑, IL-4↓, IL-10↓, IL-13↓, HRG↑, PIGF↓, Autophagy activation, CD8^+^↑, TLR4↑, CTX↑, JSI-124↑	Enhanced anti-tumor response Facilitating chemotherapy
Doxil nanomedicine and Tranilast	IFNγ↑ LPS↑	M1-type macrophage promotion Facilitating chemotherapy
Paclitaxel	TLR4↑	

*Up arrow: elevated levels or activity*.

*Down arrow: decreased levels or inhibition*.

## TAMCs in Radiotherapy

**Figure 2:**
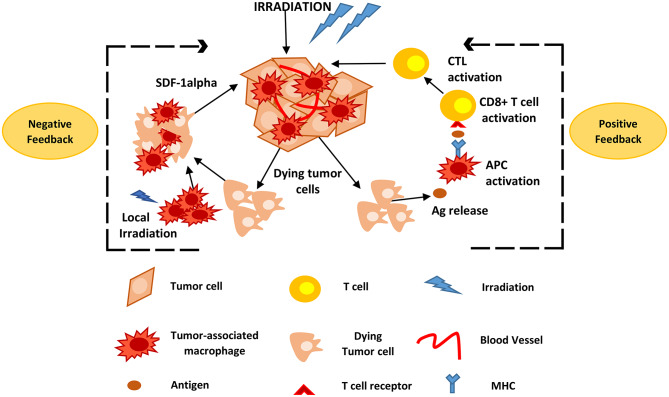
Positive and negative feedback loops of TAM activity during radiotherapy. Following high and low irradiation protocols, antigens are released from dying tumor cells and taken up by APCs, such as TAMs, that subsequently activate CD8^+^ T-cells. This causes CTL recruitment and activation that attack solid tumors. Local irradiation may also cause the accumulation of macrophages to the tumor site that promote tumor recurrence mainly *via* the expression of SDF-1alpha. Ag, Antigen; APCs, antigen presenting cells; CTL, cytotoxic T lymphocytes; TAMs, tumor associated macrophages; SDF-1alpha, stromal cell-derived factor-1 alpha.

Radiotherapy is an important and commonly used treatment approach in cancer; local radiotherapy allows for non-invasive, site-specific intervention. Even though the main mechanism of action is *via* tumor cell DNA damage, recent evidence suggests that irradiation activates tumor-specific immunity (Figure 2). The effects of radiotherapy include the induction of antigen release from dying tumor cells, the activation of APCs and the support of tumor-specific T-cell immigration and function (110–113). In a study using the RIP1-Tag5 (RT5), human melanoma xenografts mouse model, and human pancreatic cancer specimens derived from patients undergoing low-dose irradiation (LDI) of 0.5 Gy, researchers showed that neoadjuvant local LDI causes the CTL recruitment and activation in solid tumors. This was associated with the accumulation of iNOS-positive (iNOS^+^) macrophages and led to prolonged survival in xenotransplant mouse tumor models (114). Dendritic cells contribute to the immune response following high dose radiation. Local high-dose irradiation (10 Gy) leads to activation of tumor-associated DC that induce tumor-specific effector CD8^+^ T-cells (115).

Myeloid cells may also negatively affect radiotherapy. In a prostate cancer animal model, irradiation with a local daily dose of 3 Gy for 5 days led to a systemic increase of MDSCs in lymph nodes, lung, spleen, and peripheral blood and a 2-fold increase in CSF-1 in tumors. Blockade of CSFR1 by a selective inhibitor, decreased macrophage migration and in combination with radiotherapy repressed tumor growth more effectively than irradiation alone (116). In a similar approach, following radiotherapy of mammary tumor-bearing mice using localized gamma irradiation (5 Gy), the blockade of CSF-1 using a neutralizing monoclonal antibody (mAb) or a small molecule inhibitor against the CSF-1 receptor kinase (PLX3397), caused depletion of macrophages and significantly inhibited tumor growth. This was associated with increased numbers of CD8^+^ T-cells in tumors, and reduced the number of CD4^+^ T-cells, the main source of the Th2 cytokine IL4 which can lead to a pro-tumor advantage (117). Following local irradiation with 21 Gy in breast and lung carcinoma xenograft models, myeloid bone marrow-derived cells (BMDCs), primarily macrophages, rapidly accumulated in tumors. The levels of SDF-1alpha/CXCL12, a chemokine that promotes BMDCs retention in the tissue, were increased in the tumor, 2 days after local irradiation. Concurrent treatment with radiation and an inhibitor of SDF-1alpha receptor (AMD3100) significantly hindered tumor regrowth. These results suggest that macrophages promote tumor recurrence following radiation *via* increase in the expression of SDF-1alpha (118).

The contradicting reports concerning the role of myeloid cells on the efficacy of radiotherapy, may be attributed to the radiation dose and fractionation methodology. Both these parameters appear to affect the tumor microenvironment and the immune system. The conventional standard dose fractionation of 2 Gy per fraction is mostly used to achieve cell damage within the tumor (119). However, several pre-clinical studies suggest hypofractionated high doses of 6 or 8 Gy are more effective compared to a single high dose of radiation in inducing pro-immunogenic effects (120–122). *In vitro* studies have also shown that larger doses of radiation induce immunogenic cell death (ICD) (123). The effects of radiation of the TME should also be taken into account when trying to evoke an immune response. High single-fraction doses (8–16 Gy) induce increased permeability and apoptosis in endothelial cells (124). Even though a definitive radiotherapy regimen that effectively manipulates the TME and activates the immune system against the tumor has not been established, low, standard and high doses as well as different fractionation approaches have been found to be effective. It is also possible that optimized dosing and fractionation protocols may be suitable for different types and/or stages of cancer. The effects or radiation on the immune system and how it may promote tumor survival or destruction are detailed in a recent review (125).

## Role of MDSCs, TAMs and DCs in Immunotherapy

The main challenge of tumor immunologists is to control the vicious cycle of inflammation-immunosuppression taking place within the TME. The main approaches followed include among others, targeting immune checkpoint molecules on myeloid cells, the inhibition of recruitment and survival of myeloid cells, while novel approaches of nanomedicine regulating MDSCs are also under investigation.

### Targeting Immune-Checkpoint Molecules on MDSCs, TAMs, and DCs

**Figure 3:**
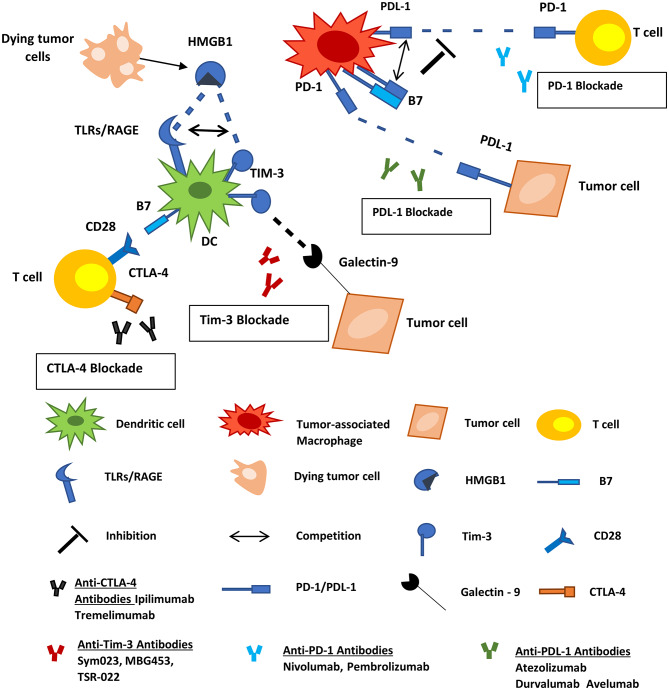
Inhibitory immune checkpoint molecules represent targets for cancer therapy. PD-1, PD-L1, TIM-3, and B7 molecules are expressed by subsets of myeloid-derived cells. PD-1 on macrophages interacts with PD-L1 on tumor cells and allows cancer progression by promoting escape from immune surveillance. TAMs also express PD-L1 and B7 molecules that can interact with the PD-1 on T-cells inhibiting the function of the latter. TIM-3 on infiltrating DCs binds to HMGB1 derived from dying tumor cells blocking anti-tumor immune responses. Tumor cells also express Galectin-9 which interacts with TIM-3 on DCs negatively regulating their function. Tumor Associated Macrophages; DCs, dendritic cells; HMGB1, high-mobility group protein 1; TLR, toll-like receptor; RAGE, receptor for advanced glycation end products.

Immune checkpoints are among the regulators of the immune system that defend self-tolerance. Various tumor cells utilize these regulators to evade immune responses (126). Inhibitory immune-checkpoint molecules, including the cytotoxic T-lymphocyte-associated protein 4 (CTLA-4), programmed cell death protein 1 (PD-1) and its ligand (PD-L1), T-cell immunoglobulin and mucin-domain containing molecule (TIM-3), ligands belonging to the B7 family, and others are promising targets for novel cancer immunotherapeutics. Antibodies against these inhibitory molecules are being tested in clinical trials, for their potential as mono- or part of combinatorial therapy against human neoplasias. Some of them have received approval by the Food and Drug Administration (F.D.A.) and entered the clinical routine practice for the therapeutic management of certain human tumors. These include (a) the human anti-CTLA-4 mAb ipilimumab for the treatment of metastatic melanoma, (b) the human anti-PD-1 mAbs nivolumab and pembrolizumab for the treatment of melanoma and unresectable/metastatic solid tumors, respectively, and (c) the anti-PD-L1 mAb atezolizumab for patients with metastatic non-small cell lung cancer (127). PD-1, PD-L1, TIM-3, and B7 molecules are expressed by subsets of TAMs and DCs, and consist therapeutic targets facilitating the inhibition of the function of these cells and the subsequent elimination of the tumor (Figure 3).

As a response to hypoxia and specific cytokines, TAMs express elevated levels of CTLA-4 ligands and other immune-checkpoint inhibitors. CTLA-4 ligands such as B7 molecules are also highly expressed in DCs of the tumor microenvironment (128). This overexpression is associated with the downregulation of anti-tumor activities of T-cells, by inhibition of the co-stimulatory interaction with CD28, both in humans and in animal models (129–132). The use of anti-CTLA-4 antibodies would hamper this inhibition and promote T-cell co-stimulation *via* CD80/CD86–CD28 interaction. A subject of debate, however, is whether the anti-CTLA-4 antibodies act in favor of anti-tumor immune responses or not, in terms of targeting the CTLA-4 molecules expressed on regulatory T-cells (Tregs). CTLA-4 expression by Tregs comprise one their main cell contact-dependent mechanisms affecting antigen-presentation to T-cells. However, it seems that anti-CTLA-4 therapy favors the blockade of the inhibitory activity of CTLA-4 on both effector T-cells and Tregs (133). Indeed, ipilimumab administration in mice bearing melanoma tumors, amplified CD8^+^ T-cell activities against tumor cells and inhibited immunosuppressive functions of Tregs (126, 133, 134). The latter is achieved *via* Treg phagocytosis by TAMs expressing Fcγ receptors (133). In a murine model of head and neck squamous cell carcinoma (HNSCC), the blockade of CTLA-4 was correlated with reduced numbers of MDSCs and M2 macrophages, and enhancement of T-cell activation in both tumor microenvironment and macro-environment (132). In humans, it has been reported that patients with advanced melanoma receiving ipilimumab exhibited significantly decreased counts of MDSCs and significantly increased counts of CD8^+^ effector/memory T-cells in the circulation (135). In addition to that, in another recent study, the percentage of FcγRIIIA^+^ CD16^+^ peripheral blood monocytes was found to be higher in melanoma patients who respond to ipilimumab therapy compared to the non-responders (136). The above data underline the pivotal role of the CD8^+^ T/MDSC balance in the outcome of anti-tumor immune responses, and more importantly how this balance can be affected by treatment with anti-CTLA-4 antibodies. Additionally, a recent study supports that dual therapy with ipilimumab and the vitamin A derivative all-trans retinoic acid (ATRA), leads to a more pronounced reduction in circulating MDSCs compared to the anti-CTLA4 monotherapy (137). ATRA is the standard-of-care treatment for patients with acute promyelocytic leukemia (APL), where it induces terminal differentiation of immature myelocytic tumor cells leading to their death (138). In a similar way, ATRA promotes the differentiation of MDSCs, resulting in their decreased numbers and function (139). Based on these properties, combinatorial administration of ATRA and ipilimumab consists a promising enhanced weapon in the treatment against human cancers.

PD-1 is expressed by a subset of macrophages and DCs of the TME. This molecule may interact with PD-L1 on tumor cells leading to the negative regulation of TAMs and DCs. Expression of PD-1 by TAMs increases with the progression of tumor in mice and in advanced stages of the disease in humans, while it is negatively associated with their phagocytic activities against tumors cells (140, 141). In murine models of cancer, inhibition of the PD-1/PD-L1 interaction enhances macrophage phagocytosis, suppresses the growth of the tumor and prolongs the survival of animals (140). Another recent study showed that the results of anti-PD-L1 therapy in tumor-bearing mice was totally abolished in PD-L1-deficient animals, supporting the importance of blocking the PD-1/PD-L1 pathway as an anti-cancer therapeutic approach (142). Despite the promising evidence from pre-clinical studies, in human clinical trials the administration of nivolumab (anti-PD-L1) in patients with renal cell carcinoma, non-small cell lung cancer or metastatic melanoma with PD-L1-negative tumors was associated with reduced clinical response (NCT00730639; *clinicaltrials.gov*) (143, 144). Tumor-infiltrating DCs and TAMs also express PD-L1 and B7 molecules that can interact with the PD-1 on T-cells obstructing the function of the latter (145). Thus, it is essential that all the above mentioned evidence are carefully considered during the development of anti-cancer immunotherapeutic strategies to act in favor of anti-tumor immunity and not by promoting its inhibition. Future clinical studies are needed to evaluate the possible beneficial impact of this blockade in patients with specific types of tumors (141).

Certain myeloid cell subsets express TIM-3, which can bind to the phosphatidylserine (PS) revealed by apoptotic cells further contributing to their presentation to CD8^+^ T-cells (146). DCs express TIM-3 which can also bind to HMGB1 derived from dying cells (147). HMGB1 and nucleic acids from apoptotic cells stimulate anti-tumor immunity, and TIM-3 can block this stimulation by competition for binding to the HMGB1-nucleic acid complex (147). Tumor cells also express galectin-9 which interacts with TIM-3 on tumor-infiltrating DCs regulating their function. Indeed, it was recently shown that anti-Tim-3 blocking in combination with Paclitaxel administration amplified anti-tumor immune responses against breast cancer *in vivo*, and this inhibition was facilitated by the galectin-9-Tim-3 interaction, rather than the HMGB1-nucleic acid-Tim3 or PS-Tim3 interaction (148). This interplay between tumor cells and tumor-infiltrating DCs is probably pivotal for the development and perpetuation of other types of malignancies (149), since galectin-9 is highly expressed by several tumor cell types (150).

Nowadays, numerous phase II and III clinical trials are testing the potential of antibodies against CTLA-4 (ipilimumab, tremelimumab), or the PD-1/PDL-1 axis (nivolumab, pembrolizumab, atezolizumab, durvalumab, avelumab), and also the combinatorial use of anti-PD-1/anti-CTLA-4 antibodies (nivolumab plus ipilimumab) in a series of human malignancies. Moreover, novel antibodies, not yet FDA approved, targeting TIM-3 immune checkpoint (including INCAGN02390, Sym023, MBG453, TSR-022) are now being tested in phase I and II trials (127).

### Inhibition of Recruitment and Survival of MDSCs

Cancer cells often express increased levels of the chemokine CCL2; also known as monocyte chemoattractant protein 1, MCP1), which recruits MDSCs (expressing the CCR2) in the site of tumor inflammation (151). Blocking the CCL2-CCR2 interaction could be another alternative to prevent the accumulation of these cells in the TME. Inhibition of this pathway has provided with promising results in murine models of pancreatic (50), hepatocellular (152) and prostate (153) cancer. Moreover, a phase II clinical trial using carlumab (anti-CCL2 mAb) in individuals with metastatic castration-resistant prostate cancer (NCT00992186; *clinicaltrials.gov*) supports that the withdrawal of anti-CCL2 treatment may lead to a rebound of CCL2 levels; in the participants of this study there was an upregulation of the CCL2 serum levels that exceeded those before treatment (154). Also, cessation of anti-CCL2 treatment was shown to accelerate metastasis in a murine model of breast cancer (155). Thus, therapeutic interventions against CCL2-CCR2 interaction need to be critically examined and future studies to evaluate their overall therapeutic efficiency.

Recent evidence supports that the interaction between CD200-CD200 receptor (CD200R) is essential for the control of immune responses in TME by regulating TAMCs. In humans, certain tumor cell types including melanoma cells (156), ovarian cancer cells (157), malignant B cells (158) and cells from some neuroendocrine neoplasms overexpress CD200. Within the TME, significantly high levels of CD200 can be also detected on endothelial cells, activated T and B cells, Tregs (159), as well as MDSCs, TAMs and DCs (160). The CD200 and CD200R molecules share structural similarities with the PD-PDL1 and CTLA4-B7 molecules and the CD200-CD200R axis is also considered as an immune-checkpoint regulator of tumor-related immune responses (161). The complicated network of interactions among these cell types can drive the outcome of immune responses in the TME and impact tumor progression.

The blockade of CD200-CD200R interactions is currently amongst the immunotherapeutic alternatives under investigation. Studies on hu-SCID (severe combined immunodeficiency) mice with established tumors (162–164) have shown that adoptively transferred peripheral blood mononuclear cells together with blockade of CD200 can lead to the rejection of the tumors. Nevertheless, there is a debate regarding the beneficial effects that the blockage of CD200-CD200R axis may have in cancer patients (141), since following treatment with chemotherapeutic agents such as doxorubicin, the recruitment of functional DCs in the TME claims the CD200-CD200R pathway (165). In humans, the anti-CD200 mAb Samalizumab has entered two phase I clinical trials: one on patients bearing solid tumors (NCT02987504) and the other on patients with B-cell chronic lymphocytic leukemia (B-CLL) or multiple myeloma (NCT00648739) (*clinicaltrials.gov*). Both studies were terminated, but the second one published results showing that administration of the drug was associated with reduced expression of CD200 on B-cells and CD4^+^ effector T-cells of B-CLL individuals, however inefficient in the three myeloma patients evaluated (166). What is more, it is expected that the use of CD200-depletion antibodies would have considerable side-effects, since CD200 is also expressed on normal cells. Therefore, the alternative way of targeting the CD200R, which is expressed by cancer but not by normal cells, would be more feasible for use in the clinical setting for the treatment of human malignancies. A study on mice with CD200-negative melanoma tumors showed that treatment with an agonistic anti-CD200R mAb inhibited the tumor formation and metastasis in the lungs of the animals, *via* inhibition of myeloid cell functions (167). A recent study on animals with colon cancer, suggests that co-treatment with anti-CD200R and a Toll-Like Receptor 7 (TLR-7) agonist promotes the anti-tumor effects of myeloid cells within the TME (168).

## Future Perspectives: Nanomedicine Approaches to Deplete, Modulate or Recruit MDSCs or TAMs

Novel approaches for the enhancement of anti-cancer therapeutics also lie in the field of nanomedicine: Based on the enhanced permeability and retention (EPR) effect therapeutic strategies that use carrier materials of < 100 nm can enhance the uptake of chemotherapeutics specifically by tumor cells, thus lowering non-specific cytotoxicity (169, 170). In the field of cancer immunotherapy, the efforts focus on the use of nanoparticles to drive various immunoregulators to tumors and recruit myeloid-derived cells to the site of tumor inflammation (169).

MDSCs and TAMs can facilitate the use of nanoparticles in anti-cancer immunotherapeutics due to their phagocytic ability (171). It was shown that in tumor-bearing mice peripherally administered with nanoparticles, the monocytic and polymorphonuclear MDSCs were preferentially targeted uptaking 10-fold more of these carrier materials compared to the tumor cells (172). Other *in vivo* studies on mice with tumors or hematological malignancies have shown that intradermal administration of nanoparticles carrying the chemotherapeutic agents 6-thioguanine or a gemcitabine derivative were accumulated in macrophages and myeloid cells of the spleen and the tumors and finally led to the depletion of the MDSC compartments in these sites, promoting adoptive T-cell therapy (172–174).

Apart from depletion, nanoparticles have been used for the polarization of MDSCs to an anti-tumor immune phenotype, using stimulants of the innate immune system, such as TLR ligands (175, 176). A recent article describes how nanoparticles loaded with R848, a TLR7/8 agonist, can promote the polarization of TAMs toward an M1 phenotype, resulting in the control of the tumor growth and protection of the animals against tumor re-challenge (177). Interestingly, the co-administration of R848-nanoparticles with anti-PD1 therapy abolished the resistance of mice to anti-PD1 treatment and led to improved response rates (177). The approach of using nanoparticles carrying mimetics of “danger signals” to induce innate anti-tumor responses together with immune checkpoint inhibitors has already entered trials in the clinical setting: nanomaterials with a TLR9 agonist and the anti-PD-1 mAb pembrolizumab are now being tested in a phase Ib/II clinical study in patients with various metastatic solid tumors [NCT03684785; *clinicaltrials.gov*].

An alternative of reprogramming macrophages using small interfering RNAs (RNAi) or micro RNAs (miRNAs) that are loaded on nanoparticles was applied to mice with melanoma, colon carcinoma, non-small cell lung cancer and other tumors, leading to encouraging results (178–180). Recently, a research group used nanoparticles that have co-encapsulated both a chemoattract of MDSCs, the CCL2 chemokine, and an RNAi sequence interfering with *Cebpb*, critical for the immunosuppression phenotypes of these cells. The administration of capsules co-carrying these two protein- and RNA- factors, induced the attraction of MDSCs while reduced the differentiation of monocytes to macrophages in *in vitro* studies of primary MDSCs and in *in vivo* experiments on fibrosarcoma mice (181). Lastly, using ferumoxytol, an FDA-approved iron supplement composed of dextran-coated iron oxide nanoparticles, Zanganeh et al. (182) managed to inhibit tumor growth by inducing the pro-inflammatory M1 phenotype in the TME of early breast tumors, and liver metastases in mice with lung cancer.

## Conclusions

It is unambiguously accepted that immune cells in the tumor stroma exert fundamental effects not only on cancer development and disease progression but also for treatment efficacy. In particular, myeloid cells residing in the tumor microenvironment, including MDSCs, TAMs, DCs and tumor-associated neutrophils (TANs), can either enhance tumor rejection or facilitate cancer progression based on their functional interplay with cancer cells. Moreover, while the variety of tumor treatment options gradually increase, including targeted therapies, nanotherapies and immunotherapies, reaching optimal levels of efficacy often appears to be hindered by the infiltration and complex interactions with myeloid cells. It is therefore critically important for future studies to further subcategorize immune cells of myeloid origin based on their pro- or anti-tumor properties. Moreover, it may be appropriate to tailor conventional treatment approaches, such as chemotherapy, nanotherapy and radiotherapy, in terms of dosing, fractionation and scheduling in order to achieve optimal conditions for activation of anti-tumor immune responses. Finally, identification and validation of exclusive cell surface marker panels for each subpopulation as well as better understanding the common pro-tumor traits of these cells will allow for better stratification of cancer patient prognosis and the development of more effective therapeutic interventions.

## Author Contributions

CN wrote the paper and helped with illustrations. T-CK wrote part of the paper, prepared illustrations. CP wrote part of the paper. M-IC wrote part of the paper and helped with illustrations. PC wrote part of the paper. PP conceived the theme, wrote the paper, and prepared illustrations. All authors critically read and approved the manuscript.

## Conflict of Interest

The authors declare that the research was conducted in the absence of any commercial or financial relationships that could be construed as a potential conflict of interest.

## Abbreviations

ATP: adenosine triphosphate
APC: antigen-presenting cell
APL: acute promyelocytic leukemia
ATRA: all-trans retinoic acid
BM: bone marrow
BMDCs: bone marrow-derived cells
B-CLL: B-Cell chronic lymphocytic leukemia
CCL-2: C-C motif chemokine ligand 2
CRC: colorectal carcinoma
CMPs: common myeloid progenitor cells
COX: cyclooxygenase
CDA: cytidine deaminase
CSF-1: colony-stimulating factor 1 receptor
CSF-1R: colony-stimulating factor-1
CSF-2: colony-stimulating factor-2
CXCL12: C-X-C motif chemokine ligand 12
CXCL5: C-X-C motif chemokine ligand 5
CTX: cyclophosphamide
CTL: cytotoxic T lymphocyte
DCs: dendritic cells
EPR: enhanced permeability and retention
ErbB1: epidermal growth factor receptor 1
ErbB2: epidermal growth factor receptor 2
ERK: extracellular signal-regulated kinase
FDA: food and drug administration
GIST: gastrointestinal stromal tumor
GEM: gemcitabine
GM-CSF: granulocyte macrophage colony-stimulating factor
g-MDSCs: granulocytic MDSCs
G-CSF: granulocyte colony-stimulating factor
HSC: hematopoietic stem cell
HNSCC: head and neck squamous cell carcinoma
HMGB1: high mobility group box 1
ICIs: immune checkpoint inhibitors
iNOS: inducible nitric oxide synthase
IDO: indoleamine 2,3-dioxygenase
IL-10: interleukin-10
IL-10R: interleukin-10 receptor
IL-2: interleukin-2
IL-4: interleukin-4
IL-6: interleukin-6
IBC: inflammatory breast cancer
IL-6R: interleukin-6 receptor
LDI: lipopolysaccharide (LPS)low-dose irradiation
MMP-9: matrix metalloproteinase 9
MTD: maximum tolerated dose
MAPK: mitogen-activated protein kinase
M-CSF: macrophage colony-S factor
MHC: major histocompatibility complex
MMPs: matrix metalloproteinases
MITF: microphthalmia transcription factor
MTX: mitoxantrone
MDSCs: myeloid-derived suppressor cells
MEFs: murine embryonic fibroblasts
NPs: nanoparticles
NO: nitric oxide
NK cells: natural killer (NK) cells
NSCLC: non-small cell lung cancer
OS: overall survival
PTX: paclitaxel
PDAC: pancreatic ductal adenocarcinoma
PS: phosphatidylserine
PI3K: phosphatidylinositol-3-kinase
PlGF: placental growth factor
pDC: plasmacytoid dendritic cells
PMNs: polymorphonuclear neutrophils
PGE2: prostaglandin E2
PD-1: programmed cell death protein 1
PD-L1: programmed cell death protein ligand 1
RAGE: receptor for advanced glycation end products
ROS: reactive oxygen species
Tregs: regulatory T-cells
STAT1: signal transducer and activator of transcription 1
STAT3: signal transducer and activator of transcription 3
STAT6: signal transducer and activator of transcription 6
SDF-1alpha: stromal-serived factor 1 alpha
TCR: T-cell receptor
TLR4: toll-like receptor 4
TNPs: therapeutic nanoparticles
TTR: time-to-relapse
C/EBP: transcriptional regulator CCAAT/enhancer-binding protein
TGF-β: transforming growth factor-β
TME: tumor microenvironment
TNFα: tumor necrosis factor-α
TAMs: tumor-associated macrophages
TAMCs: tumor-associated myeloid cells
TANs: tumor-associated neutrophils
TILs: tumor-infiltrating lymphocytes
VEGF: vascular endothelial growth factor
ZA: zoledronic acid.
